# Comparison of visual performance between monofocal and multifocal intraocular lenses of the same material and basic design

**DOI:** 10.1038/s41598-020-72473-x

**Published:** 2020-09-23

**Authors:** Hirotaka Tanabe, Hitoshi Tabuchi, Tomohiro Shojo, Tomofusa Yamauchi, Kosuke Takase

**Affiliations:** 1 Department of Ophthalmology, Tsukazaki Hospital, Himeji, Japan; 2 Department of Technology and Design Thinking for Medicine, Hiroshima University Graduate School of Biomedical and Health Sciences, Hiroshima, Japan

**Keywords:** Medical research, Eye diseases, Lens diseases

## Abstract

To compare the visual performance of a monofocal intraocular lens (IOL) (ZCB00) and a multifocal IOL (ZMB00) of the same material and basic design, we evaluated postoperative parameters at 10 weeks after the last surgery in cataract patients who underwent bilateral ZCB00 or ZMB00 implantation from December 13, 2010, to July 29, 2019, with the right and left lenses implanted within 3 months of each other. The study enrolled 2,230 eyes of 1,115 patients. The monofocal group comprised 904 eyes of 452 patients (72.3 ± 6.8 years; females/males, 268/184), and the multifocal group comprised 1,326 eyes of 663 patients (67.0 ± 7.8 years; females/males, 518/145). Contrast sensitivity (4.0/2.5/1.6/1.0/0.7 degrees), contrast sensitivity with glare (1.6/1.0/0.7 degrees), and the VFQ-25 score for driving at night were significantly better in the monofocal group (p < 0.00068, Wald test). Uncorrected intermediate/near visual acuity and near spectacle independence were significantly better in the multifocal group (p < 0.00068, Wald test). The two IOL groups had different characteristics in terms of contrast sensitivity, night-time driving, uncorrected intermediate/near visual acuity and near spectacle independence.

## Introduction

There is a long-standing debate about whether monofocal or multifocal lenses should be implanted during cataract surgery.

Multifocal lenses have been shown to provide good distance and near functional vision without the use of corrective lenses^1–8^ and have been improved in many ways. However, many studies have reported that multifocal lenses compromise contrast sensitivity and night-time driving with unwanted subjective phenomena, such as halos, glare and starbursts^9–13^, which could affect the patient’s visual performance and satisfaction. Those reports have inhibited many Japanese from choosing multifocal lenses, and a national survey in 2019 reported that the rate of multifocal IOL use for cataract surgery in Japan was only 3.9%^14^.

However, to achieve the ultimate goals of cataract surgery, i.e., fast and complete visual rehabilitation and optimal satisfaction regarding vision-related issues, multifocal lenses have continually evolved over the years and achieved better outcomes^15^, and the range of choices is much larger now than in the past.

The Tecnis monofocal IOL (ZCB00) and the Tecnis multifocal IOL (ZMB00) are representative IOLs that have been used in Japan for more than a decade because of the high quality of the materials, as shown in previous reports; these lenses have an aspherical, modified prolate anterior surface that is designed to minimize spherical aberrations and improve contrast sensitivity under mesopic conditions after cataract surgery^16–18^. Except for additional bifocal diffraction gratings in the multifocal IOLs with + 4.0 dioptres, they have the same design as clear acrylic 6.0-mm optics.

In our previous study, we retrospectively compared the visual performance of two small groups of patients who received monofocal IOLs (ZCB00) (170 eyes of 85 patients) or multifocal IOLs (ZMB00) (92 eyes of 46 patients) in cataract surgery at the cataract unit of the Department of Ophthalmology of Tsukazaki Hospital between April 2009 and January 2012^19^. In the current study, we obtained larger numbers of patients who received these lens types (ZCB00 monofocal IOLs, 904 eyes of 452 patients; ZMB00 multifocal IOLs, 1,326 eyes of 663 patients) from December 13, 2010, to July 29, 2019.

In this study, we retrospectively compared the visual performance of the Tecnis monofocal IOL (ZCB00) and the Tecnis multifocal IOL (ZMB00) based on the accumulated data of a decade of practice at a single eye institute.

## Results

### Patient characteristics

The patient demographics and pre/postoperative visual parameters are shown in Supplementary Table S1. The study enrolled 2,230 eyes of 1,115 patients. The monofocal group comprised 904 eyes of 452 patients (72.3 ± 6.8 years; females/males, 268 [59.3%]/184 [40.7%]), and the multifocal group comprised 1,326 eyes of 663 patients (67.0 ± 7.8 years; females/males, 518 [78.1%]/145 [21.9%]).

### Comparison of postoperative parameters between Tecnis monofocal IOLs (ZCB00) and Tecnis multifocal IOLs (ZMB00)

**Figure 1:**
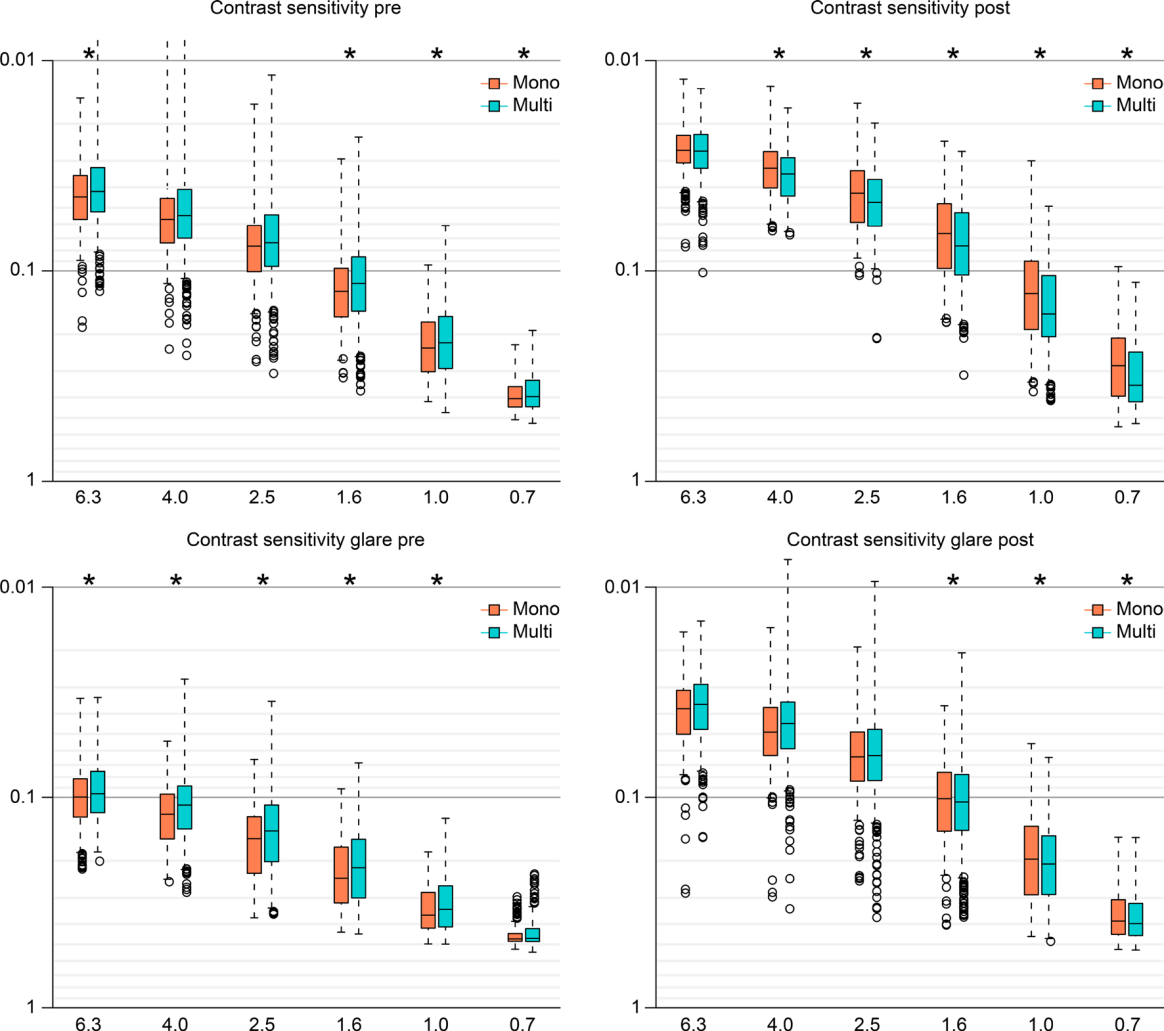
Contrast sensitivity with or without glare in the monofocal and multifocal groups before and 10 weeks after surgery on both eyes. The solid line connects the median values for each group. Each parameter was adjusted by multiple linear regression with the explanatory variables in Table 1. The significance level was set to 0.0083 after Bonferroni’s correction.

**Figure 2:**
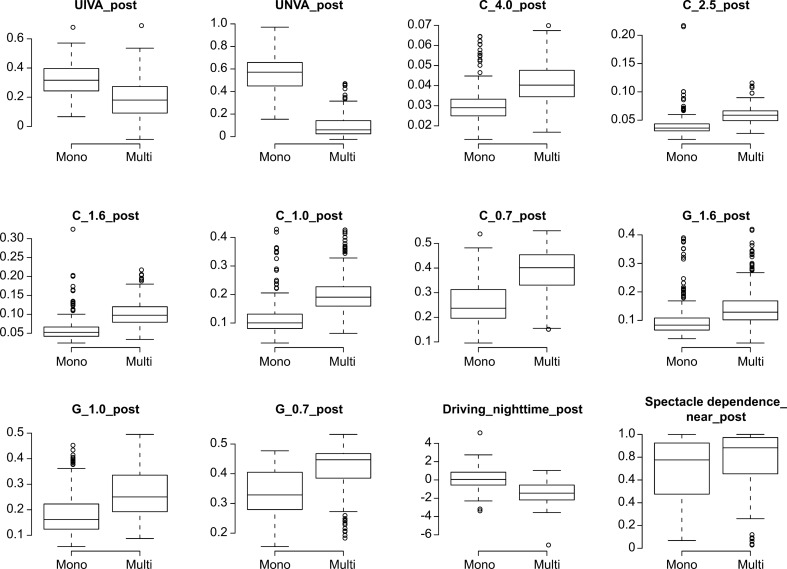
Parameters that demonstrated a significant difference between the monofocal and multifocal groups at 10 weeks after surgery on both eyes. Each parameter was adjusted by multiple regression with the explanatory variables in Table 1.

Multiple regression analysis was applied to all postoperative parameters of the monofocal and multifocal groups at 10 weeks after surgery on both eyes; the parameters were adjusted by multiple regression with the explanatory variables in Table 1, and the results of the analysis are shown in Supplementary Table S2. Contrast sensitivity (4.0/2.5/1.6/1.0/0.7 degrees), contrast sensitivity with glare (1.6/1.0/0.7 degrees), and the Visual Function Questionnaire (VFQ)-25 score for driving at night were significantly better in the monofocal group (p < 0.00068, Wald test), and corrected near visual acuity, contrast sensitivity (6.3 degrees), contrast sensitivity with glare (2.5 degrees), and the VFQ-25 scores for mental health/driving in general/driving during the day/driving in adverse conditions were likely better in the monofocal group (p < 0.05, Wald test) (Table 2, Figs. 1, 2). Uncorrected intermediate/near visual acuity and near spectacle independence were significantly better in the multifocal group (p < 0.00068, Wald test), and the VFQ-25 score for general health and distance spectacle independence were likely better in the multifocal group (p < 0.05, Wald test) (Table 2, Fig. 2).

**Table 1:** Parameters in the monofocal and multifocal groups used to adjust the linear regression model: age, sex, axial length (at the time of surgery), subjective refraction spherical equivalent (SE), subjective refraction cylinder (CYL), corneal astigmatism (keratometric cylinder), corneal higher-order aberrations (astigmatism, total higher-order aberration (HOA), third, fourth, trefoil, coma, tetrafoil, second-order astigmatism (2ndAstig), and spherical, scaled to a pupil size of 4 mm/6 mm).

Variable	Levels	N (%)	Multi	p value (Wald test)
**(A) Categorical variable**				
Sex	F/M	268 (59.3)/184 (40.7)	518 (78.1)/145 (21.9)	2.020E−11*

Variable	N, Mean ± SD	Multi	p value (Wald test)	
	Mono			
**(B) Continuous variables**				
Age	452, 72.301 ± 6.817	663, 67.043 ± 7.809	6.078E−56*	
SE	762, 0.110 ± 0.474	1,069, 0.229 ± 0.448	2.263E−09*	
CYL	640, − 0.955 ± 0.535	849, − 0.785 ± 0.397	1.167E−08*	
Corneal astigmatism	754, − 0.780 ± 0.461	525, − 0.731 ± 0.413	1.940E−01	
Axial length	900, 23.536 ± 1.283	1,326, 24.045 ± 1.574	1.728E−13*	
**WF_4_post_C**				
Astigmatism	581, − 0.998 ± 0.631	953, − 0.873 ± 0.501	3.497E−03*	
Total HOA	581, 0.233 ± 0.127	953, 0.204 ± 0.105	1.051E−06*	
Third	581, 0.201 ± 0.123	953, 0.174 ± 0.100	8.894E−06*	
Fourth	581, 0.107 ± 0.059	953, 0.098 ± 0.054	1.202E−03*	
Trefoil	581, 0.155 ± 0.102	953, 0.131 ± 0.086	1.163E−05*	
Coma	581, 0.112 ± 0.092	953, 0.100 ± 0.075	3.852E−02*	
Tetrafoil	581, 0.069 ± 0.047	953, 0.059 ± 0.045	1.951E−06*	
2nd Astig	581, 0.047 ± 0.042	953, 0.039 ± 0.030	1.577E−03*	
Spherical	581, 0.045 ± 0.045	953, 0.048 ± 0.047	4.554E−02*	
**WF_6_post_C**				
Astigmatism	500, − 0.740 ± 1.021	868, − 0.623 ± 0.433	3.277E−03*	
Total HOA	500, 0.704 ± 1.285	868, 0.580 ± 0.435	1.932E−11*	
Third	500, 0.474 ± 0.894	868, 0.390 ± 0.310	4.443E−06*	
Fourth	500, 0.440 ± 0.719	868, 0.368 ± 0.249	4.180E−14*	
Trefoil	500, 0.348 ± 0.636	868, 0.278 ± 0.236	7.559E−06*	
Coma	500, 0.288 ± 0.646	868, 0.245 ± 0.235	1.005E−01	
Tetrafoil	500, 0.212 ± 0.466	868, 0.169 ± 0.180	1.957E−06*	
2nd Astig	500, 0.134 ± 0.457	868, 0.100 ± 0.158	1.727E−02*	
Spherical	500, 0.321 ± 0.345	868, 0.274 ± 0.162	3.986E−07*	
Pupil diameter post	592, 4.190 ± 0.811	980, 4.480 ± 0.871	2.523E−10*	

For categorical data, each category and its count and frequency are shown, and Fisher’s exact test was used to compare categorical data for the monofocal and multifocal IOLs. For numerical data, the mean and standard deviation are shown, and the Mann–Whitney test was used to compare numerical data for the monofocal and multifocal IOLs.

*SE* subjective refraction spherical equivalent, *CYL* subjective refraction cylinder, *WF_4_post_C_* wavefront_4_post_corneal, *HOA* higher-order aberration.

*p < 0.05.

**Table 2:** Parameters that demonstrated a significant difference at p < 0.00068 or p < 0.05 between the monofocal and multifocal groups at 10 weeks after surgery on both eyes.

Response_post	After adjustment		Coefficient (95% CI)	p value (Wald test)
	Mono	Multi		
UIVA	0.33 ± 0.12	0.20 ± 0.13	− 0.14 (− 0.20, − 0.09)	1.541E−06**
UNVA	0.56 ± 0.16	0.09 ± 0.10	− 0.47 (− 0.51, − 0.43)	2.6E−16**
CNVA	0.02 ± 0.03	0.02 ± 0.05	0.01 (0.00, 0.03)	1.859E−02*
**Contrast sensitivity**				
C_6.3	0.03 ± 0.01	0.03 ± 0.01	0.00 (0.00, 0.01)	1.215E−02*
C_4.0	0.03 ± 0.01	0.04 ± 0.01	0.01 (0.01, 0.02)	1.214E−10**
C_2.5	0.04 ± 0.02	0.06 ± 0.01	0.02 (0.01, 0.03)	1.860E−09**
C_1.6	0.06 ± 0.03	0.10 ± 0.03	0.04 (0.03, 0.05)	2.726E−16**
C_1.0	0.12 ± 0.06	0.20 ± 0.07	0.10 (0.08, 0.11)	2.6E−16**
C_0.7	0.26 ± 0.08	0.39 ± 0.08	0.14 (0.12, 0.17)	2.6E−16**
**Contrast sensitivity with glare**				
G_2.5	0.07 ± 0.05	0.08 ± 0.04	0.02 (0.00, 0.03)	2.387E−02*
G_1.6	0.10 ± 0.06	0.14 ± 0.07	0.04 (0.02, 0.06)	9.586E-−**
G_1.0	0.18 ± 0.08	0.27 ± 0.10	0.09 (0.07, 0.12)	3.476E−11**
G_0.7	0.34 ± 0.08	0.42 ± 0.07	0.09 (0.07, 0.12)	1.929E−11**
**VFQ-25**				
General health	57.42 ± 4.89	64.76 ± 3.77	5.00 (1.63, 8.36)	3.602E−03*
Mental health	94.84 ± 2.85	91.14 ± 3.27	− 3.59 (− 6.01, − 1.17)	3.614E−03*
Driving, general	86.91 ± 5.38	81.51 ± 5.46	− 5.50 (− 10.22, − 0.78)	2.228E−02*
Driving, daytime (low/high)	10/40	62/5	− 1.24 (− 2.11, − 0.37)	5.407E−03*
Driving, night-time (low/high)	12/34	52/15	− 1.26 (− 1.94, − 0.59)	2.379E−04**
Driving, adverse conditions (low/high)	13/34	44/24	− 0.61 (− 1.19, − 0.03)	4.053E−02*
**Spectacle dependence**				
Distance	66/0/17	88/0/2	− 2.49 (− 4.16, − 0.82)	3.539E−03*
Near	14/2/67	87/1/3	− 6.26 (− 8.00, − 4.51)	2.260E−12**

Each parameter was adjusted by multiple regression with the explanatory variables in Table 1. For each response variable, the mean and standard deviation for each numerical parameter or the counts for each categorical parameter (Spectacle dependence: never/sometimes/always), the regression coefficient, its 95% confidence interval, and the p value (Wald test) are shown.

*UIVA* uncorrected intermediate visual acuity, *CIVA* corrected intermediate visual acuity, *UNVA* uncorrected near visual acuity, *CNVA* corrected near visual acuity, *C* contrast sensitivity, *G* contrast sensitivity under glare.

*p < 0.05, **p < 0.00068.

### Correlation among postoperative parameters of Tecnis monofocal IOLs (ZCB00) and Tecnis multifocal IOLs (ZMB00)

**Figure 3:**
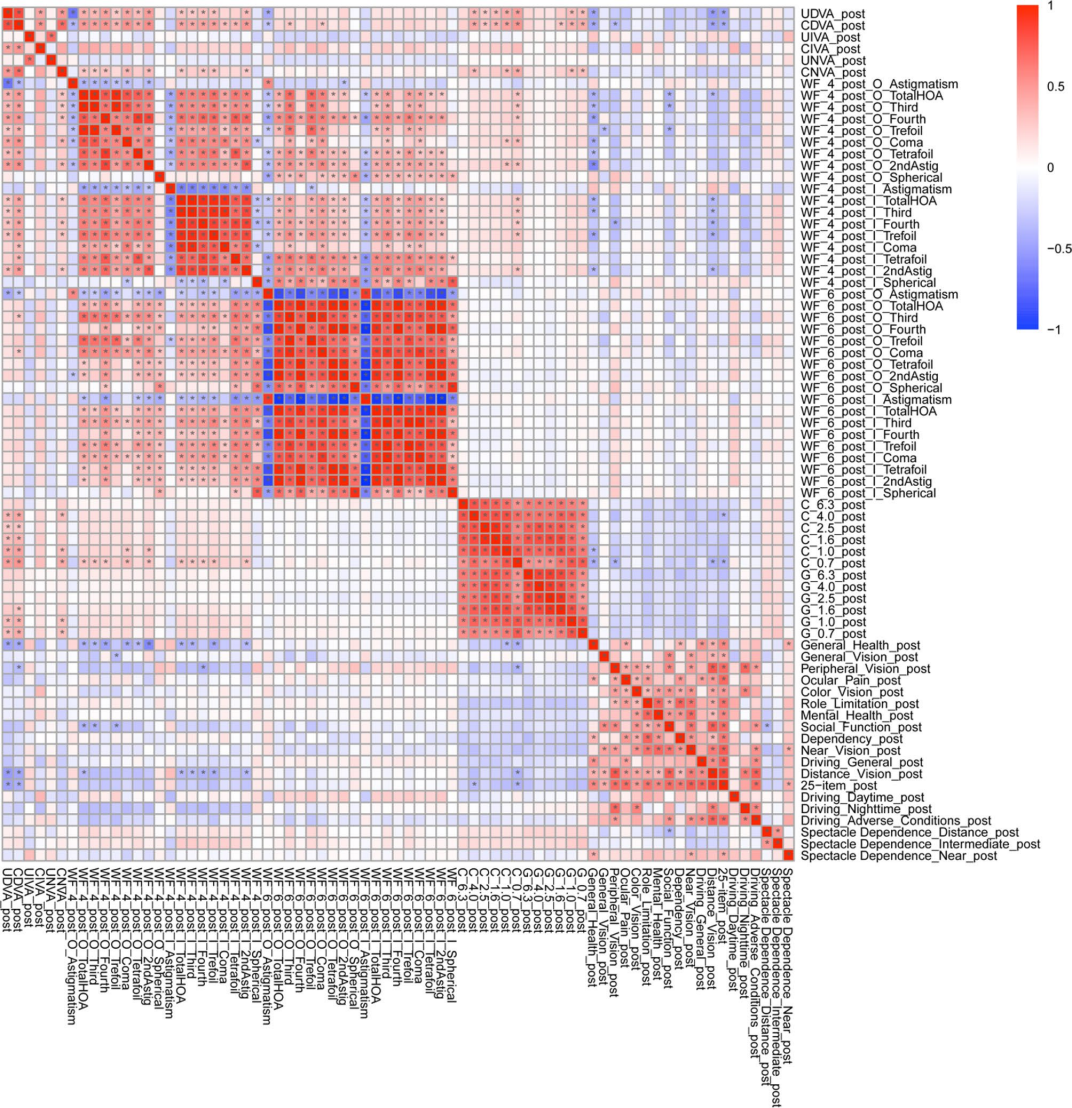
Heatmap of correlation coefficients between all possible combinations of variables, which were adjusted by multiple regression with the explanatory variables in Table 1, in the monofocal IOL group. The asterisk * in this figure indicates a significant correlation between two parameters at p < 0.00002 after Bonferroni’s correction. The illustration was performed using a commercially available software program (R, version 3.6.1; R Core Team, 2019, Vienna, Austria.)^59^ (https://cran.r-project.org/web/packages/pheatmap/pheatmap.pdf).

**Figure 4:**
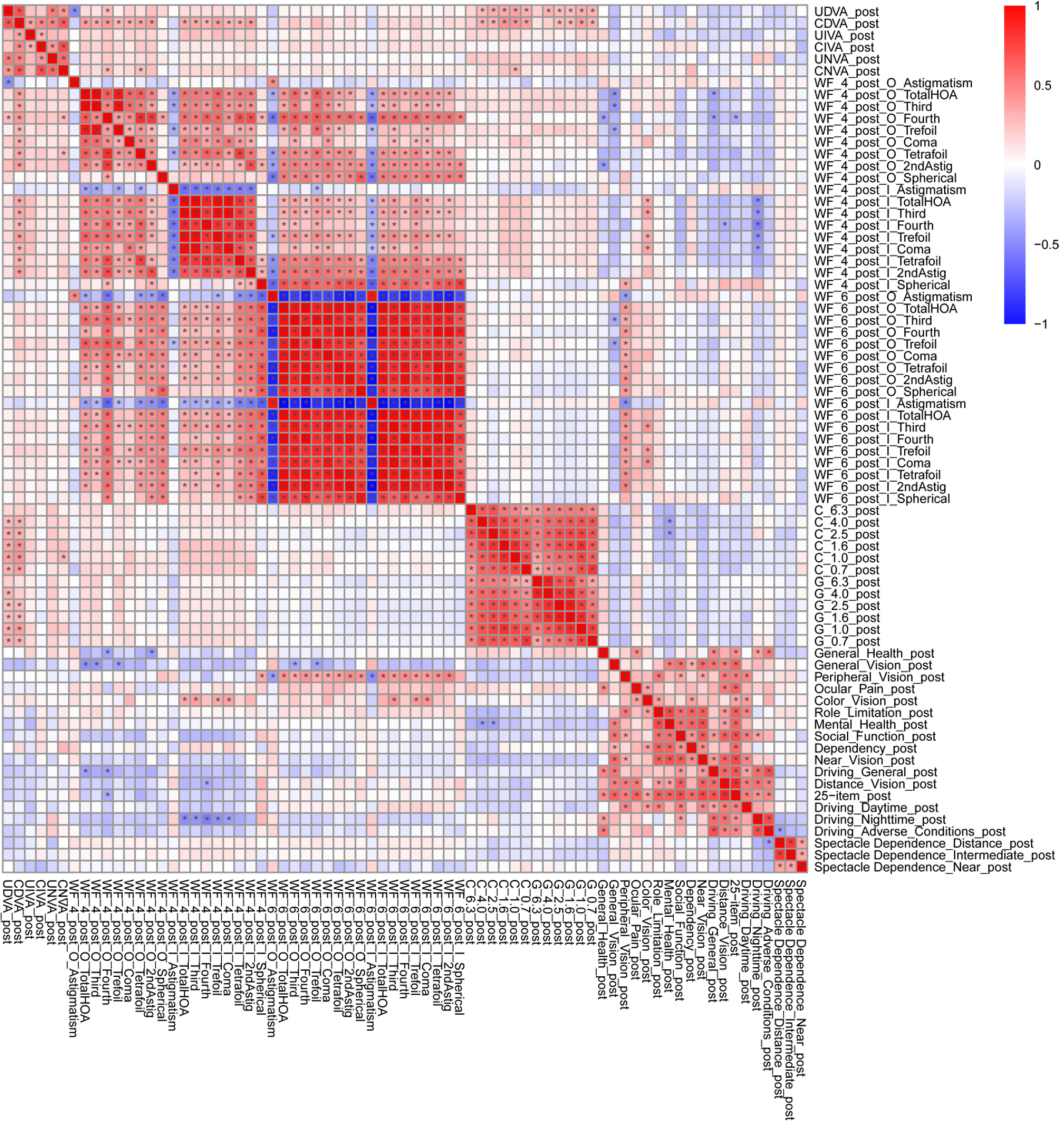
Heatmap of correlation coefficients between all possible combinations of variables, which were adjusted by multiple regression with the explanatory variables in Table 1, in the multifocal group. The asterisk * in this figure indicates a significant correlation between two parameters at p < 0.00002 after Bonferroni’s correction. The illustration was performed using a commercially available software program (R, version 3.6.1; R Core Team, 2019, Vienna, Austria)^59^ (https://cran.r-project.org/web/packages/pheatmap/pheatmap.pdf).

The correlation coefficients (A) and p values for the correlation analyses (B) between all possible combinations of postoperative parameters for the monofocal and multifocal groups were adjusted by multiple regression with the explanatory variables in Table 1 and are shown in Supplementary Table S3, Fig. 3, Supplementary Table S4, Fig. 4, respectively.

## Discussion

We previously reported the results of a retrospective study in which we compared the visual performance of two small groups of patients who received monofocal IOLs (ZCB00) (170 eyes of 85 patients) or multifocal IOLs (ZMB00) (92 eyes of 46 patients)^19^. In that study, we showed that patients in the multifocal IOL group demonstrated better uncorrected near visual acuity and lower spectacle dependence (intermediate/near), and those in the monofocal IOL group had better CIVA/CNVA and higher VFQ-25 scores for night-time driving. In the current study, we obtained a larger number of samples based on data accumulated over a decade of practice at a single eye institute. The superior uncorrected intermediate visual acuity in the multifocal group and the lack of a significant difference in CIVA/CNVA and intermediate spectacle dependence between the two groups were novel results that build upon our previous report. Although many studies using modern multifocal IOLs have reported a noninferior CNVA in multifocal groups compared to monofocal groups^20,21^, a tendency toward better CNVA in the monofocal group is suggested again by the present study, although the results were not statistically significant. Diffractive multifocal IOLs divide light into two foci; Tecnis multifocal IOLs use 41% of incoming light for distance vision and 41% for near vision, regardless of the pupil diameter, whereas the remaining 18% is lost to higher-order scattering^22,23^. The 41% of light used for distance vision allows photopic, high-contrast distance acuity comparable to that provided by monofocal IOLs, as confirmed by recent studies^2,24–26^. The Array SA40N (AMO), a first-generation multifocal IOL, was a major step towards the safe and effective treatment of presbyopia and cataracts and achieved high levels of patient satisfaction^13,27–32^. However, a report demonstrated that even with the addition of near focus, the AMO provides poorer near contrast sensitivity than can be achieved by an appropriate monofocal near correction, regardless of the spatial frequency or illumination conditions, presumably due to the pupil constriction as a result of accommodation^33^ and the IOL characteristics^24^. The ZMB00 used in the present study is a second-generation multifocal IOL with an aspherical IOL design, which improves contrast sensitivity by reducing or cancelling the normal positive spherical aberration of the cornea, the performance of which is less dependent on pupil size. Aspherical IOLs have shown decreased wavefront spherical aberrations and improved contrast sensitivity compared with spherical IOLs^16,34–38^, and an aspherical multifocal IOL design thus reduces the incidence and severity of halos and glare, which are inherent in the design (i.e., edges of the steps of different ring zones) of diffractive multifocal IOLs and are seen more often with spherical multifocal IOLs. Likewise, improved quality of vision is also seen with monofocal aspherical IOL designs, such as that of the ZCB00 used in the present study^39^. Thus, we compared the two types of new-generation aspherical IOLs, and it was difficult to determine which lens is better or worse. We concluded that at a high level of performance, they have different characteristics in terms of various visual parameters.

We used the CGT1000 instrument to measure contrast sensitivity. In this method, patients are asked whether they can distinguish any changes in the brightness contrast of a circular optotype of variable size consisting of three coloured concentric circles. This device can measure contrast sensitivity at 6 sizes and 13 contrast levels with or without glare^40^. In this study, the contrast sensitivity measured with the CGT1000 was better in the monofocal group at most frequencies both with and without glare (Figs. 1, 2), a finding that is consistent with the findings of previous reports^18,41,42^. Although contrast sensitivity can theoretically deteriorate with the use of diffractive multifocal IOLs, our results indicate better contrast sensitivity, both with and without glare, in patients in the multifocal group than in the normal 60-year-old Japanese subjects evaluated by Takahashi^43^, as stated in our previous report^19^. In both the monofocal IOL and multifocal IOL groups, the heatmaps of the correlation coefficients between all possible combinations of variables, which were adjusted by multiple regression with the explanatory variables in Table 1, demonstrated that contrast sensitivity (with/without glare) and UDVA/CDVA/CNVA were strongly correlated with each other (Figs. 3, 4). In other words, contrast sensitivity (with/without glare) is suggested to play a very important role in UDVA/CDVA/CNVA. The tendency toward superior CNVA in the monofocal group might be due to the superior contrast sensitivity (with/without glare) in this group.

Approximately 82.2% of patients in the multifocal group were perfectly spectacle independent in our study, which is consistent with the finding of 85% in our previous report^18^ and the findings of other study reports on Tecnis multifocal IOLs, in which the percentage ranged from 82.6 to 92.8%^2,20,42,44^. In contrast, the rate of spectacle independence in the monofocal group was approximately 10.8% in our study. Near spectacle independence was significantly higher and distance spectacle independence was likely higher in the multifocal group. Multifocal glasses are a very common solution for presbyopia in Japan; thus, patients who undergo implantation with monofocal IOLs often use multifocal glasses after cataract surgery. This may help explain the higher ratio of distance spectacle dependence of patients in the monofocal IOL group, whose UDVA was sufficient.

The NEI VFQ-25 is a questionnaire used for scoring the self-reported, vision-targeted health status of people with chronic eye diseases^45,46^. It has been used in normal subjects as well as those with ocular diseases, such as age-related macular degeneration, cataracts, glaucoma, and Graves’ ophthalmopathy^45,47–52^. Changes in the NEI VFQ-25 score have been reported after surgery for cataracts, glaucoma, primary vitreous floaters, vitreomacular adhesion and macular holes^53–57^. The NEI VFQ-25 has been translated into several languages (including Italian, French, German, Spanish, Turkish, Chinese, Greek, Portuguese, Arabic, and Serbian), and the Japanese version of the NEI VFQ-25 was validated by Suzukamo et al.^58^. In our study, the VFQ-25 score for driving at night was significantly better in the monofocal group, and the VFQ-25 scores for driving in general, driving during the day, and driving in adverse conditions were likely better in the monofocal group. The actual VFQ-25 scores for driving at night, driving in general, driving during the day, and driving in adverse conditions in the monofocal and multifocal groups, however, showed no compromise, even in the multifocal group. Interestingly, the VFQ-25 score for mental health was likely better in the monofocal group, while the VFQ-25 score for general health was likely better in the multifocal group. The heatmap of the correlation coefficients in the monofocal IOL group demonstrated that the VFQ-25 score for general health significantly correlated with UDVA/CDVA, contrast sensitivity, and most ocular/internal higher-order aberrations scaled to a pupil size of 4 mm (Fig. 3). On the other hand, the heatmap of the correlation coefficients showed that the VFQ-25 score for mental health significantly correlated with contrast sensitivity in the multifocal group (Fig. 4). Considering the lack of significant correlations of the VFQ-25 score for mental health with UDVA/CDVA and any ocular/internal higher-order aberrations scaled to a pupil size of 4 mm in both IOL groups (Figs. 3, 4), we can conclude that different characteristics affect the factors related to these psychological evaluation items.

Alio et al.^21^ reported that the VFQ-25 scores for near vision are significantly better in patients with multifocal IOLs than in those with monofocal IOLs and that there are no significant differences in scores for night-time driving between the two groups. We hypothesize that the probable reasons for these discrepancies from our results are differences in the monofocal IOLs used in the study, i.e., Alio evaluated the Acri.Smart 48S (Carl Zeiss Meditec AG), a single-piece, spherical, foldable, acrylic IOL, and we evaluated aspherical monofocal IOLs, which achieve improved contrast sensitivity by reducing or cancelling the normal positive spherical aberration of the cornea and the incidence and severity of halos and glare. As mentioned above, two types of second-generation IOLs were compared with each other at a high level of performance. National differences in attitudes towards eyewear could also be a contributing factor. All of the subjects in our study were Japanese patients who had little psychological resistance against wearing glasses, especially those patients who opted for the implantation of monofocal IOLs. In Japanese culture, glasses are quite common, and many people do not feel inconvenienced if they must use glasses to read books or newspapers. We consider this phenomenon to partially explain why the VFQ-25 score for near vision of patients in the monofocal group was not inferior to that of patients in the multifocal group, who had better UNVA and UIVA and a higher rate of spectacle independence.

In conclusion, we compared the visual performance of monofocal IOLs and multifocal IOLs of the same material and basic design. Patients in the multifocal group had better uncorrected intermediate/near visual acuity and higher spectacle independence, whereas patients in the monofocal group had better contrast sensitivity and higher scores for night-time driving. The superior uncorrected intermediate visual acuity in the multifocal group and the lack of a significant difference in CIVA/CNVA and intermediate spectacle dependence between the two groups were novel results that build upon our previous report. At a high performance level, both IOL groups had different characteristics in terms of various visual parameters.

## Methods

### Design

Retrospective comparative case series.

### Setting

Ophthalmology, Tsukazaki Hospital, Japan.

### Patients

We reviewed the cases of cataract patients who underwent bilateral implantation of Tecnis monofocal IOLs (ZCB00) or Tecnis multifocal IOLs (ZMB00) from December 13, 2010, to July 29, 2019, with the right and left lenses implanted within 3 months of each other. The exclusion criteria were a history of other ocular diseases that could affect visual function, |subjective equivalent (SE)|> 2.00 dioptres, |subjective refraction cylinder (CYL)|> 3.00 dioptres and |corneal astigmatism (keratometric cylinder)|> 3.00 dioptres at 10 weeks after surgery.

### Preoperative examination

Preoperatively, all patients received full ophthalmologic examinations, including evaluations of the corneal curvature radius, corneal astigmatism, axial length, refractive status, ocular aberrations, pupil diameter, distance/intermediate/near visual acuity, contrast sensitivity, and contrast sensitivity under glare, as well as anterior segment evaluations using a slit lamp, tonometry and indirect fundoscopy. The quality of vision was evaluated using the Japanese version of the 25-item National Eye Institute Visual Function Questionnaire (NEI VFQ-25)^58^. The NEI VFQ-25 was administered by experienced technicians or nurses in a face-to-face setting. Spectacle use was also evaluated by inquiring how often the patient used spectacles for distance, intermediate and near vision (with possible responses of ‘never,’ ‘sometimes’ or ‘always’).

Uncorrected distance visual acuity (UDVA) and corrected distance visual acuity (CDVA) were measured at 5.0 m. Uncorrected intermediate visual acuity (UIVA) and corrected intermediate visual acuity (CIVA) were measured at 0.5 m. Uncorrected near visual acuity (UNVA) and corrected near visual acuity (CNVA) were measured at 0.3 m. Visual acuity was measured using the decimal visual acuity chart, and the measured decimal values were converted to the logarithm of the minimum angle of resolution (logMAR) scale. The corneal curvature radius, corneal astigmatism and objective refractive status were measured using a KR-8900 autorefractor keratometer (Topcon, Tokyo, Japan). The axial length was measured using IOL Master (Carl Zeiss, Oberkochen, Germany) and AL-3000 (TOMEY, Nagoya, Japan) biometers. Contrast sensitivity and contrast sensitivity under glare were measured using a CGT-1000 contrast glare tester (Takagi Seiko, Nakano, Japan), and the pupil diameter and ocular aberrations were measured using a KR-1W Wavefront Analyzer (Topcon, Tokyo, Japan). All measurements were obtained by experienced technicians.

### IOLs and surgical technique

The patients chose to undergo implantation with either monofocal or multifocal IOLs after they had been informed of the advantages and disadvantages associated with each type. Patients in the monofocal group received Tecnis monofocal IOLs (ZCB00) bilaterally, while those in the multifocal group received Tecnis multifocal IOLs (ZMB00) bilaterally. ZCB00 and ZMB00 have an aspherical, modified prolate anterior surface designed to minimize spherical aberrations and improve contrast sensitivity under mesopic conditions after cataract surgery^16–18^. Except for the additional bifocal diffraction gratings in multifocal IOLs with + 4.0 dioptres, they have the same design: both are clear acrylic optics measuring 6.0 mm in diameter.

Cataract surgeries were performed by 19 experienced cataract surgeons using the same standard technique of sutureless microincision phacoemulsification and the same protocol. The surgical procedures consisted of topical anaesthesia, the creation of a scleral or corneal incision of 1.8 to 2.8 mm, 5 mm of continuous capsulorhexis, phacoemulsification cataract extraction and IOL implantation with an injector.

### Postoperative examination

Patients were evaluated at 10 weeks postoperatively. The postoperative examination protocol at 10 weeks was identical to the preoperative protocol.

### Statistical analyses

The two groups (monofocal IOL and multifocal IOL) were compared in terms of the following postoperative parameters at 10 weeks after surgery for both eyes: (1) mixed-effects linear regression: visual acuity (uncorrected/corrected, distance/intermediate/near), contrast sensitivity (with/without glare), and higher-order aberrations (ocular/internal, scaled to a pupil size of 4 mm/6 mm); (2) linear regression model or logistic regression: VFQ-25 score; and (3) cumulative logistic regression: spectacle dependence (distance/intermediate/near). Both groups were adjusted for age, sex, axial length, subjective refraction spherical equivalent, subjective refraction cylinder, corneal astigmatism, corneal higher-order aberrations and pupil diameter. In the regression analysis (2) and (3), the data were divided into two parts (left-eye data and right-eye data), and the regression model was applied to each data set. Since discrete scores were observed for “Peripheral_Vision”, “Color_Vision”, “Driving_Daytime”, “Driving_Nighttime”, “Driving_Adverse_Conditions” in VFQ-25, we treated them as binary data. We divided the patients into two groups (those with scores of 75 or lower and those with scores above 75) and applied the logistic regression model to them. The results of the left- and right-eye analyses were combined using the inverse variance method; the corrected values were calculated for the left- and right-eye datasets, and the average values were used.

In the regression analysis, the Wald test was applied to evaluate the significance of differences in postoperative parameters between the two groups, and the significance level was set to 0.00068 after Bonferroni’s correction. Correlation analysis between postoperative parameters was applied for the monofocal and multifocal groups, and a heatmap of Pearson’s correlation coefficients was generated for each group. In the correlation analysis, the t-test was used to evaluate whether the correlation coefficient was significantly different from zero, and the significance level was set to 0.00002 after Bonferroni’s correction.

The statistical analyses were performed using a commercially available software program (R, version 3.6.1; R Core Team, 2019, Vienna, Austria)^59^.

### Ethics statement

This study conformed to the tenets of the Declaration of Helsinki and was approved by the Ethics Committee of Tsukazaki Hospital. Written informed consent was obtained from each subject. This study was registered as UMIN000035630: ‘‘Performance comparison among different intraocular lenses in cataract surgery’’.

## Supplementary information

Supplementary Legends

Supplementary Table S1.

Supplementary Table S2.

Supplementary Table S3.

Supplementary Table S4.

## Data Availability

All data relevant to the study are included in this article or have been uploaded as supplementary information.
