# Author Correction: Comparison of visual performance between monofocal and multifocal intraocular lenses of the same material and basic design

**DOI:** 10.1038/s41598-020-76803-x

**Published:** 2020-11-05

**Authors:** Hirotaka Tanabe, Hitoshi Tabuchi, Tomohiro Shojo, Tomofusa Yamauchi, Kosuke Takase

**Affiliations:** 1 Department of Ophthalmology, Tsukazaki Hospital, Himeji, Japan; 2 Department of Technology and Design Thinking for Medicine, Hiroshima University Graduate School of Biomedical and Health Sciences, Hiroshima, Japan

Correction to: *Scientific Reports* 10.1038/s41598-020-72473-x, published online 23 September 2020

This Article contains an error in Figure 2, where each graph is incorrect in the panel ‘Spectacle dependence_near_post’.

The correct Figure 2 appears below as Figure 1.

**Figure 1:**
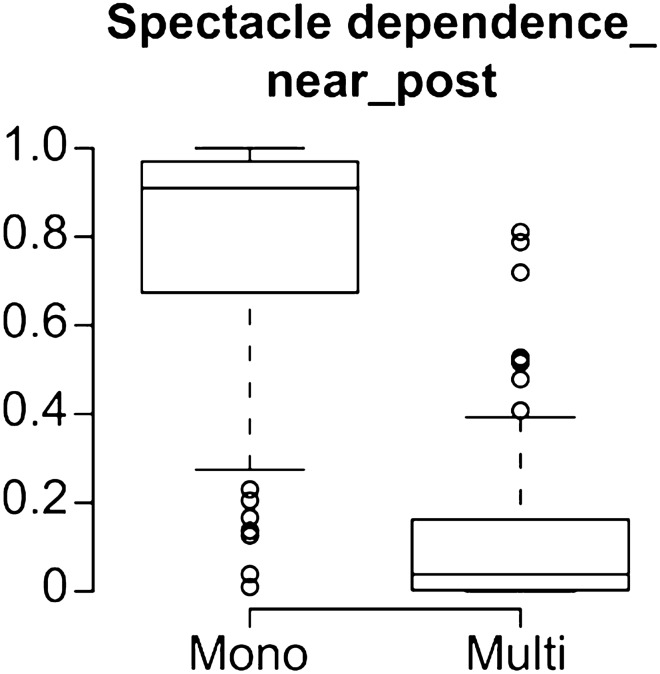
A correct version of the original Figure 2 ‘Spectacle dependence_near_post’.

