# Supplementary material for: Comparison of visual performance between monofocal and multifocal intraocular lenses of the same material and basic design
Source: Sci Rep. 2020 Sep 23;10:15490. doi: 10.1038/s41598-020-72473-x (PMC7511318; doi:10.1038/s41598-020-72473-x)
Supplement: Supplementary file 1 — Supplementary Legends [file 41598_2020_72473_MOESM1_ESM.docx]

**Supplementary Table S1.** Patient demographics and pre/postoperative visual parameters. For categorical data, each category and its count and frequency are shown, and Fisher’s exact test was used to compare categorical data for the monofocal and multifocal IOLs. For numerical data, the mean and standard deviation are shown, and the Mann-Whitney test was used to compare numerical data for the monofocal and multifocal IOLs.

**Supplementary Table S2.** Results of multiple regression analysis of all postoperative parameters of the monofocal and multifocal groups at 10 weeks after surgery on both eyes. For numerical parameters, multiple mixed linear regression or multiple linear regression was applied, and cumulative logistic regression was applied to spectacle dependence parameters. In the multiple linear regression or cumulative logistic regression, the variables in Table 1 were used as the explanatory variables. For each response variable, the regression coefficient, its 95% confidence interval, and the p value (Wald test) are shown in (A). The original and corrected values (i.e., before and after adjusting with multiple linear regression) of the mean and standard deviation for each numerical parameter and the counts for each categorical parameter (Spectacle Dependence: never/sometimes/always), regression coefficient, 95% confidence interval, and p value (Wald test) are shown in (B).

**Supplementary Table S3.** Correlation coefficients (A) and p values from the correlation analysis (B) of all possible combinations of postoperative parameters, which were adjusted by multiple regression with the explanatory variables in Table 1, in the monofocal group.

**Supplementary Table S4.** Coefficients (A) and p values from the correlation analysis (B) of all possible combinations of postoperative parameters, which were adjusted by multiple regression with the explanatory variables in Table 1, in the multifocal group.
